# HIF-1α/microRNA-128-3p axis protects hippocampal neurons from apoptosis via the *Axin1*-mediated Wnt/β-catenin signaling pathway in Parkinson’s disease models

**DOI:** 10.18632/aging.102636

**Published:** 2020-03-12

**Authors:** Guangping Zhang, Luzhu Chen, Jing Liu, Yan Jin, Zaihong Lin, Shu Du, Zenghui Fu, Tuantuan Chen, Yinghui Qin, Fenghu Sui, Yan Jiang

**Affiliations:** 1 The Fourth Ward, Department of Neurology, The Third Affiliated Hospital of Qiqihar Medical University, Qiqihar 161000, P.R. China; 2 Department of Health Care, The Third Affiliated Hospital of Qiqihar Medical University, Qiqihar 161000, P.R. China

**Keywords:** Parkinson’s disease, hypoxia inducible factor-1α, microRNA-128-3p, AXIN1, Wnt/β-catenin signaling pathway

## Abstract

Parkinson's disease (PD) is a progressive neurodegenerative disorder. A common and disabling disease of the elderly, the standard dopamine replacement therapies do not arrest the ongoing neurodegeneration, thus calling for new treatment strategies. The present study aimed to clarify the functional relevance of the hypoxia inducible factor-1α (HIF-1α)/microRNA-128-3p (miR-128-3p) axis in hippocampal neurodegeneration in a PD mouse model obtained by intraperitoneal injection of MPTP. Targeting relationship between miR-128-3p and *Axin1* was verified, so we probed the roles of *Hif1a*, miR-128-3p, and *Axin1* in apoptosis of hippocampal neurons with gain- and loss-of function experiments using flow cytometry and TUNEL staining. We found that *Axin1* was upregulated in hippocampal tissues and cells of the MPTP-lesioned mouse model of PD, while *Hif1a* and miR-128-3p were downregulated. Elevation of HIF-1α/miR-128-3p inhibited apoptosis of hippocampal neurons via Wnt/β-catenin signaling pathway activation due to the suppression of *Axin1* in PD. In addition, forced overexpression of *Hif1a* could ameliorate motor dysfunction and pathological changes in the model. Collectively, activation of the HIF-1α/miR-128-3p axis could repress hippocampal neurodegeneration in MPTP-lesioned mice through an activated Wnt/β-catenin pathway due to *Axin1* downregulation.

## INTRODUCTION

Parkinson’s disease (PD) is a common and complex neurodegenerative disorder with a high incidence in aging population across the world, resulting in severe disability due to progressive degeneration of the nigrostriatal dopaminergic pathway [1]. PD is characterized by motor symptoms including bradykinesia, postural disturbance, muscular rigidity, and dystonia as well as non-motor symptoms including sleep disturbance, cognitive deterioration, dementia, depression, and anxiety. [2]. Although PD can develop at any age, the elderly are particularly at risk, such that the incidence is 15 per 100,000 every year and the prevalence approximately 1% among individuals aged 60 years or older [3]. The development of PD is associated with alterations in multiple cellular processes, including apoptosis, oxidative stress, and mitochondrial dysfunction [4]. Although multiple risk factors, such as environment, genetic susceptibility and ageing all contribute to the occurrence and progression of PD, its pathogenesis still remains unclear [5]. Thus, there is a call for finding novel and more accurate predictors to give better diagnosis and prognosis of patients with PD.

microRNAs (miRNAs) are crucial factors in nervous system development, function and disease, and serve as potential biomarkers for neurodegenerative disorders, including PD [6, 7]. Certain miRNAs, such as miR-124 and miR-128, are highly expressed in neurons [8], and miR-128 plays an established role in PD [9]. Moreover, miR-128 is upregulated by hypoxia-inducible factor-1α (HIF-1α) in patients with glioma [10]. HIF-1α, as a leading transcription element for modulation of the cellular responses to hypoxia, is of great importance as an emerging marker for the progression of PD [11]. In addition, HIF-1α can exert therapeutic effects against some neurodegeneration via promoting cell survival signals, such as those mediated by the Wnt/β-catenin signaling pathway [12]. Wnt acts as a key signaling cascade which modulates several cellular processes in PD, including differentiation, neuronal survival, neurogenesis, and neuroprotection [13]. β-catenin, which is expressed in human brain, is a critical component of the Wnt signaling pathway [14], such that a dysregulated Wnt/β-catenin signaling pathway participates in the pathophysiology of PD [15]. Furthermore, AXIN1, negatively regulates the canonical Wnt signaling pathway [16]. The interplay between AXIN1 and the Wnt/β-catenin signaling pathway plays a central regulatory role in cell apoptosis [17–19]. Besides, *Axin1* has been proposed as a novel gene involved in PD pathogenesis [20]. Importantly, *Axin1* has been recognized as a target gene of miR-128, thus presenting a promising therapeutic candidate for PD [21]. Given the aforementioned review, we hypothesized that the HIF-1α/miR-128-3p axis might have an effect on PD pathology by regulating *Axin1* and the associated Wnt/β-catenin signaling pathway.

## RESULTS

### Screening of DEGs and prediction of upstream regulatory miRNAs in PD

The first step to calculate our results in our experiment is to investigate whether the HIF-1α/miR-128-3p axis affected hippocampal neurodegeneration by regulating *Axin1* with the help of screening of the GEO database (PD-related microarray data GSE7621) revealed that *Axin1* was one of the most upregulated DEGs in PD (Supplementary Figure 1A). Overexpression of *Axin1* is known to downregulate the Wnt/β-catenin signaling pathway, resulting in hippocampal neurodegeneration [22]. The upstream regulatory miRNAs of *Axin1* were predicted by Targetscan. There were binding sites between miR-128-3p and *Axin1* (Supplementary Figure 1B), which proved to have considerable sequence homology between human and mouse (Supplementary Figure 1C). miR-128-3p overexpression can alleviate motor disturbances in a model of PD [9], and HIF-1α can upregulate miR-128-3p, thus preventing neuronal injury [10, 23]. Thus, we inferred that the HIF-1α/miR-128-3p axis mediating *Axin1* supported hippocampal neurodegeneration via the Wnt/β-catenin signaling pathway in PD.

### *Axin1* is upregulated, while *Hif1a* and miR-128-3p are downregulated in PD

**Figure 1:**
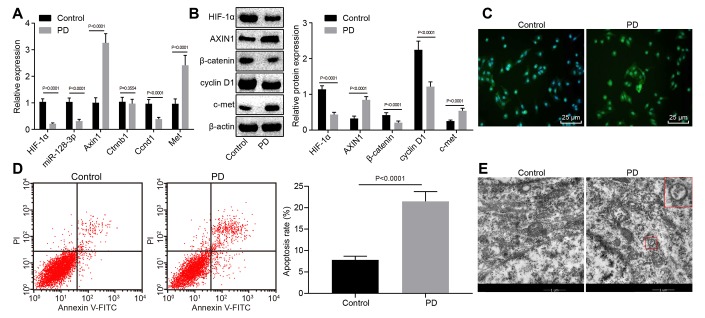
**The expression pattern of HIF-1α/miR-128-3p/AXIN1 and the Wnt/β-catenin signaling pathway-related proteins (β-catenin, cyclin D1 and c-met) in hippocampal neurons of normal mice (n = 10) and the MPTP-lesioned mouse model of PD (n = 30).** (**A**) The miR-128-3p expression and mRNA levels of *Hif1a, Axin1, Ctnnb1, Ccnd1,* and *Met* determined by RT-qPCR. (**B**) The protein levels of HIF-1α, AXIN1, β-catenin, cyclin D1, and c-met normalized to β-actin as determined by western blot analysis. (**C**) The localization of β-catenin protein in hippocampal tissues detected by the immunofluorescence assay (scale bar = 25 μm). (**D**) The hippocampal neuron apoptosis identified by flow cytometry. (**E**) The ultrastructure of hippocampal neurons through electron microscopy. * *p* < 0.05 *vs.* the control group (primary hippocampal neurons of normal mice).

Since c-met, cyclin D1 and β-catenin of the Wnt/β-catenin signaling pathway were closely associated with normal neuronal function [24, 25] and since *Axin1* promotes hippocampal neuron degeneration by downregulating the Wnt/β-catenin signaling pathway [22], we tested the expression levels of *Hif1a,* miR-128-3p, *Axin1, Ctnnb1, Ccnd1,* and *c-met* in hippocampal tissues of normal and MPTP-lesioned mice. RT-qPCR showed increased mRNA levels of *Axin1* and *Met*, but decreased miR-128-3p expression and mRNA levels for *Hif1a* and *Ccnd1* MPTP-lesioned mice (*p* < 0.05), while *Ctnnb1* mRNA level did not differ from that in normal mice (*p* > 0.05) (Figure 1A). Besides, MPTP-lesioned mice had increased protein levels of AXIN1 and c-met but reduced levels of HIF-1α, β-catenin and cyclin D1 (*p* < 0.05) (Figure 1B). An immunofluorescence assay showed that the nuclear content of β-catenin was conspicuously lower in hippocampal tissues of MPTP-lesioned mice (Figure 1C). Flow cytometry revealed a significantly increased ratio of apoptotic cells in the hippocampal tissues of MPTP-lesioned mice (Figure 1D). Ultrastructural observation with electron microscopy (Figure 1E) showed intact morphology, clear structure, normal nuclear morphology, and uniformly distributed chromatin in hippocampal neurons of normal mice, whereas MPTP-lesioned mice exhibited severe degeneration, extremely irregular nuclear morphology, lobulated indentations on the nuclear membrane, shrinkage of chromatin within the nuclei, and early apoptotic changes in the lesioned hippocampus. Thus, hippocampal tissues of MPTP-lesioned mice had upregulated *Axin1* and *Met*, downregulated *Hif1a*, miR-128-3p, and *Ccnd1*; β-catenin showed increased protein degradation and reduced nuclear translocation, but no change was found at the mRNA level.

### *Axin1* is a target gene of miR-128-3p

**Figure 2:**
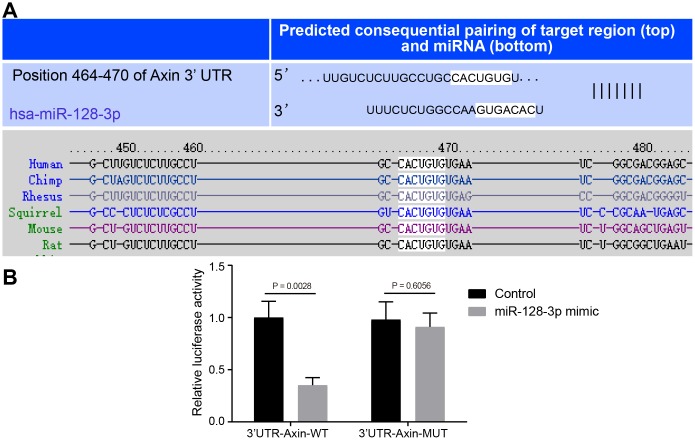
**The targeting relationship between *Axin1* and miR-128-3p.** (**A**) The binding sites between miR-128-3p and *Axin1* 3'UTR predicted by Targetscan (http://www.targetscan.org/vert_71/). (**B**) The targeting relationship between miR-128-3p and *Axin1* verified using dual-luciferase reporter gene assay. In the X-axis, 3'UTR-*Axin1*-WT refers to *Axin1* wild type 3’UTR and 3'UTR-*Axin1*-MUT refers to *Axin1* mutant 3’UTR. * *p* < 0.05 *vs.* the control group (primary hippocampal neurons of normal mice). The experiment was repeated three times independently.

The Targetscan website showed that miR-128-3p could potentially target *Axin1* (Figure 2A), so we next investigated the relationship between miR-128-3p and *Axin1* in the PD model. Dual-luciferase reporter gene assay (Figure 2B) indicated that luciferase activity of wild type of *Axin1* 3'UTR was inhibited by miR-128-3p mimic (*p* < 0.05), while that of mutant 3'UTR showed no significant difference (*p* > 0.05), suggesting that *Axin1* was inversely regulated by miR-128-3p.

### HIF-1α/miR-128-3p overexpression activates the Wnt/β-catenin signaling pathway by downregulating *Axin1* in primary hippocampal neurons of in the MPTP-lesioned mouse PD model

**Figure 3:**
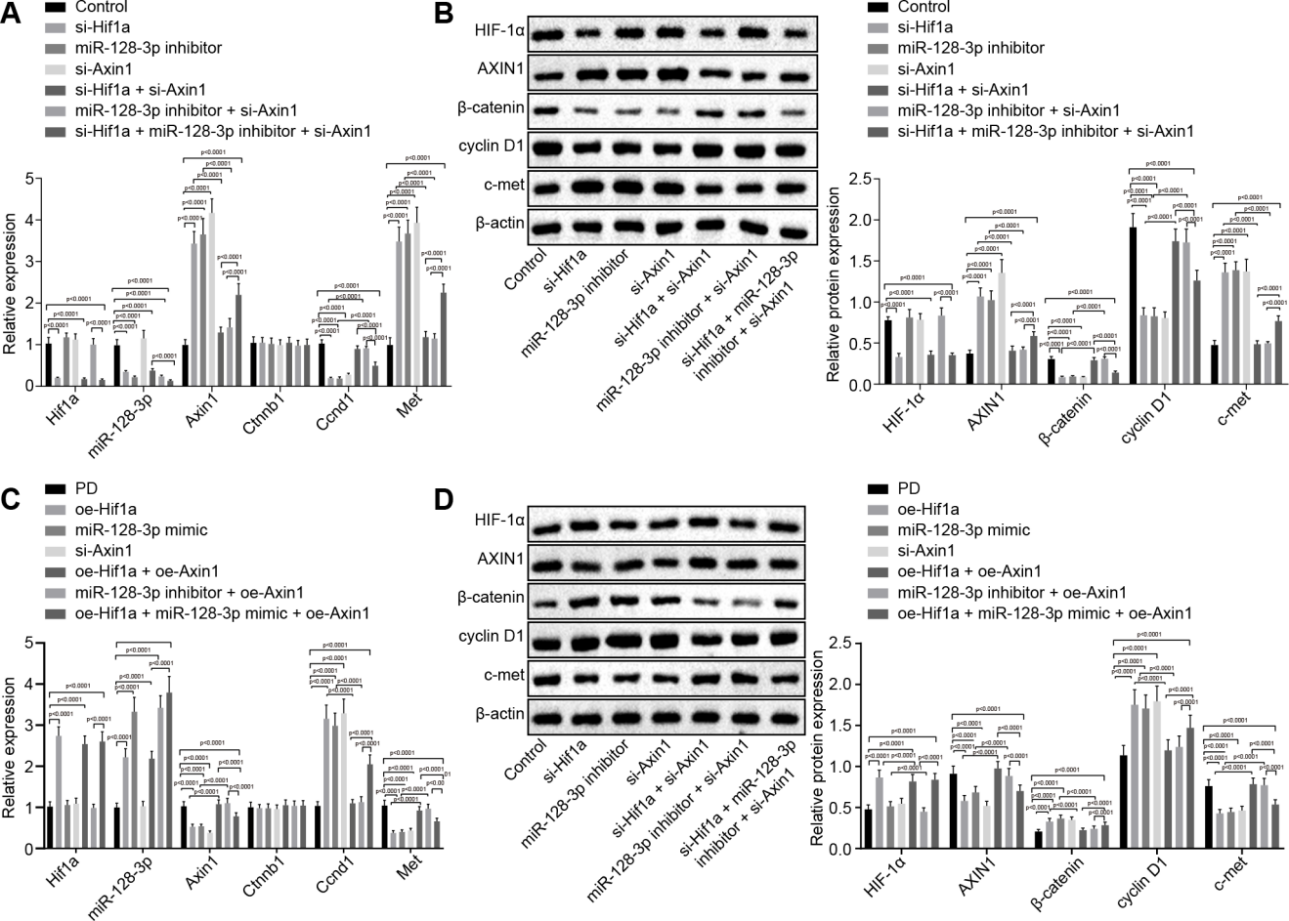
**The expression pattern of HIF-1α/miR-128-3p/AXIN1 and the Wnt/β-catenin signaling pathway-related proteins (β-catenin, cyclin D1 and c-met) in hippocampal neurons of normal mice (n = 10) and in the MPTP-lesioned mouse model of PD (n = 30) after transduction.** (**A**) The expression levels of *Hif1a,* miR-128-3p, *Axin1, Ctnnb1, Ccnd1,* and *Met* in primary hippocampal neurons of normal mice after treated with *si-Hif1a*, miR-128-3p inhibitor, or *si-Axin1*, determined by RT-qPCR. (**B**) The protein band patterns and the protein levels of HIF-1α, AXIN1, β-catenin, cyclin D1, and c-met normalized to β-actin in primary hippocampal neurons of normal mice after treatment with *si-Hif1a*, miR-128-3p inhibitor, or *si-Axin1*, all as determined by western blot analysis. * *p* < 0.05 *vs.* the control group (primary hippocampal neurons of normal mice); # *p* < 0.05 *vs.* the *si-Hif1a* group (hippocampal neurons of normal mice treated with *si-Hif1a*); & *p* < 0.05 *vs.* the miR-128-3p inhibitor group (hippocampal neurons of normal mice treated with miR-128-3p inhibitor); @ *p* < 0.05 *vs.* the *si-Hif1a* + *si-Axin1* group (hippocampal neurons of normal mice treated with *si-Hif1a* + *si-Axin1*); $ *p* < 0.05 *vs.* the miR-128-3p inhibitor + *si-Axin1* group (hippocampal neurons of normal mice treated with miR-128-3p inhibitor + *si-Axin1*). (**C**) The expression levels of *Hif1a,* miR-128-3p, *Axin1, Ctnnb1, Ccnd1,* and *Met* in primary hippocampal neurons cultured from the MPTP-lesioned mouse model of PD after treatment with *oe-Hif1a*, miR-128-3p mimic, or oe-*Axin1*, as determined by RT-qPCR. (**D**) The protein band patterns and the protein levels of HIF-1α, AXIN1, β-catenin, cyclin D1, and c-met normalized to β-actin in primary hippocampal neurons of the MPTP-lesioned mouse model of PD after treatment with *oe-Hif1a*, miR-128-3p mimic, or oe-*Axin1*, as determined by western blot analysis. * *p* < 0.05 *vs.* the PD group (primary hippocampal neurons of MPTP-lesioned mouse model of PD); # *p* < 0.05 *vs.* the *oe-Hif1a* group (hippocampal neurons of MPTP-lesioned mouse model of PD treated with *oe-Hif1a*); & *p* < 0.05 *vs.* the miR-128-3p mimic group (hippocampal neurons of MPTP-lesioned mouse model of PD treated with miR-128-3p mimic); @ *p* < 0.05 *vs.* the *oe-Hif1a* + oe-*Axin1* group (hippocampal neurons of MPTP-lesioned mouse model of PD treated with *oe-Hif1a* + oe-*Axin1*); $ *p* < 0.05 *vs.* the miR-128-3p mimic + oe-*Axin1* group (hippocampal neurons of MPTP-lesioned mouse model of PD treated with miR-128-3p mimic + oe-*Axin1*). The experiment was repeated tree times independently.

With the results in the above section detailing the relationship between miR-128-3p and *Axin1*, the focus of the experiment was shifted to investigation on the effects of the HIF-1α/miR-128-3p axis along with *Axin1* on the Wnt/β-catenin signaling pathway in primary hippocampal neurons of normal mice. RT-qPCR (Figure 3A) and western blot analysis (Figure 3B) showed decreased expression of cyclin D1 (*Ccnd1*) and increased expression of c-met (*Met*) in hippocampal neurons from mice treated with *si-Hif1a*, miR-128-3p inhibitor or oe-*Axin1* in comparison to normal hippocampal neurons (*p* < 0.05) while *Ctnnb1* mRNA expression remained unchanged (*p* > 0.05) and β-catenin protein level was relatively decreased (*p* < 0.05). Silencing *Axin1* appeared to reverse the regulatory role of HIF-1α/miR-128-3p on cyclin D1, c-met and β-catenin. Thus, HIF-1α/miR-128-3p silencing, by upregulating *Axin1* could suppress the activation of the Wnt/β-catenin signaling pathway, and silenced *Axin1* could reverse the effects of HIF-1α/miR-128-3p inhibition on the Wnt/β-catenin signaling pathway in primary hippocampal neurons of normal mice. However, delivery of *oe-Hif1a*/miR-128-3p mimic or *si-Axin1* had opposite effects in MPTP-lesioned mice, i.e. HIF-1α/miR-128-3p overexpression downregulated *Axin1*, which weakened the inhibitory effects on the activation of the Wnt/β-catenin signaling pathway, while overexpressed *Axin1* proved to reverse the effects of HIF-1α/miR-128-3p overexpression on the Wnt/β-catenin signaling pathway (Figure 3C, 3D).

### HIF-1α/miR-128-3p overexpression inhibits apoptosis of hippocampal neurons of MPTP-lesioned mouse model of PD by downregulating *Axin1*

**Figure 4:**
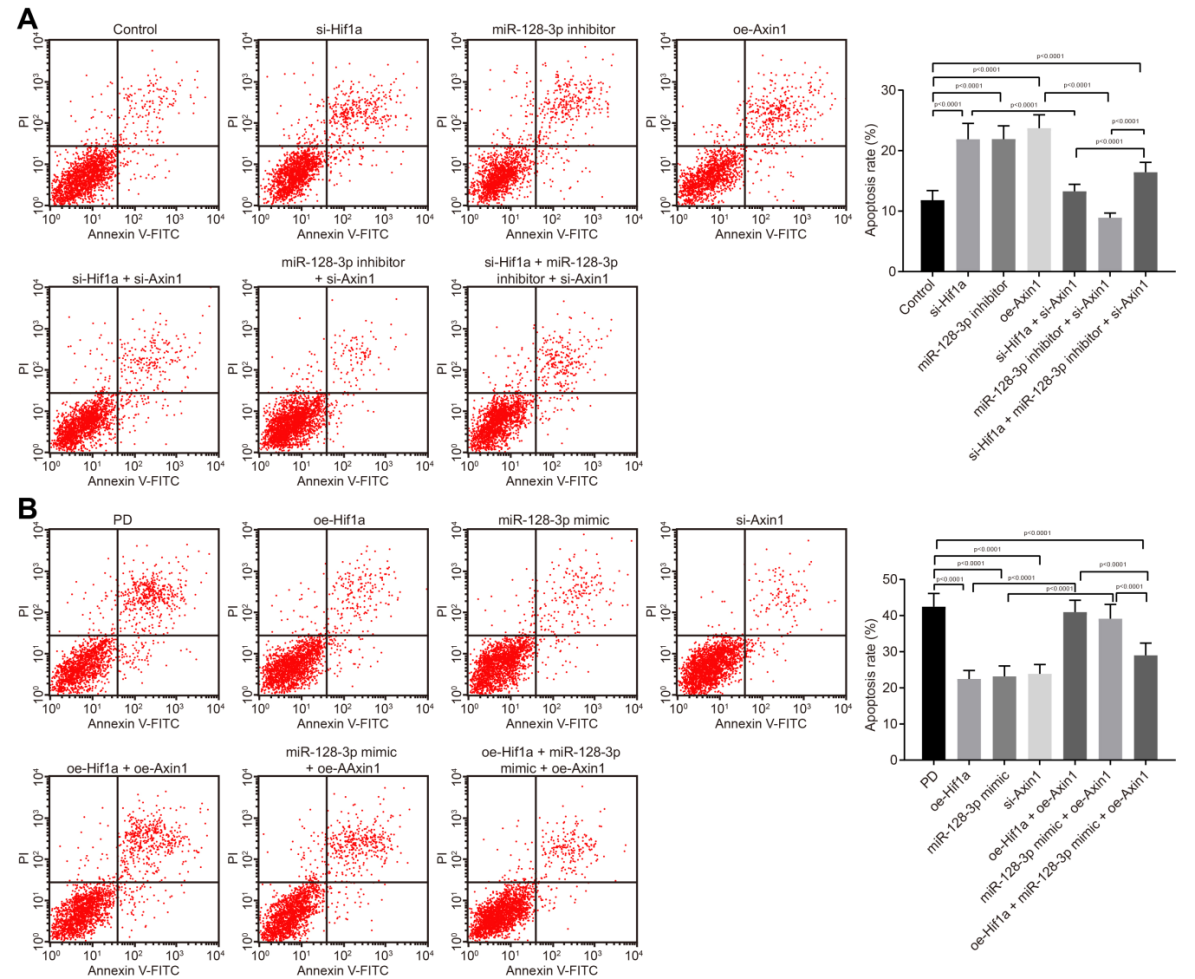
**The apoptosis of hippocampal neurons cultured from normal mice and an MPTP-lesioned mouse model of PD mediated by the HIF-1α/miR-128-3p/AXIN1 axis.** (**A**) The apoptosis of hippocampal neurons of normal mice after treatment with *si-Hif1a*, miR-128-3p inhibitor, or *si-Axin1* as detected by flow cytometry. * *p* < 0.05 *vs.* the control group (primary hippocampal neurons of normal mice); # *p* < 0.05 *vs.* the *si-Hif1a* group (hippocampal neurons of normal mice treated with *si-Hif1a*); & *p* < 0.05 *vs.* the miR-128-3p inhibitor group (hippocampal neurons of normal mice treated with miR-128-3p inhibitor); @ *p* < 0.05 *vs.* the *si-Hif1a* + *si-Axin1* group (hippocampal neurons of normal mice treated with *si-Hif1a* + *si-Axin1*); $ *p* < 0.05 *vs.* the miR-128-3p inhibitor + *si-Axin1* group (hippocampal neurons of normal mice treated with miR-128-3p inhibitor + *si-Axin1*). (**B**) The apoptosis of hippocampal neurons in the MPTP-lesioned mouse model of PD after treatment with *oe-Hif1a*, miR-128-3p mimic, or oe-*Axin1* detected by flow cytometry. * *p* < 0.05 *vs.* the PD group (primary hippocampal neurons of the MPTP-lesioned mouse model of PD); # *p* < 0.05 *vs.* the *oe-Hif1a* group (hippocampal neurons of the MPTP-lesioned mouse model of PD treated with *oe-Hif1a*); & *p* < 0.05 *vs.* the miR-128-3p mimic group (hippocampal neurons of MPTP-lesioned mouse model of PD treated with miR-128-3p mimic); @ *p* < 0.05 *vs.* the *oe-Hif1a* + oe-*Axin1* group (hippocampal neurons of MPTP-lesioned mouse model of PD treated with *oe-Hif1a* + oe-*Axin1*); $ *p* < 0.05 *vs.* the miR-128-3p mimic + oe-*Axin1* group (hippocampal neurons of MPTP-lesioned mouse model of PD treated with miR-128-3p mimic + oe-*Axin1*). The experiment was repeated three times independently.

Flow cytometry (Figure 4A) revealed that apoptosis was promoted in hippocampal neurons from mice treated with *si-Hif1a*, miR-128-3p inhibitor or oe-*Axin1* (*p* < 0.05), the effects of which tended to be reversed by silencing *Axin1*. The above results demonstrated that HIF-1α/miR-128-3p silencing facilitated apoptosis of hippocampal neurons from normal mice, and silenced *Axin1* could counteract the effects of HIF-1α/miR-128-3p in silencing apoptosis. As expected, hippocampal neuron apoptosis in MPTP-lesioned mice was significantly suppressed by overexpressed HIF-1α/miR-128-3p or silenced *Axin1* (*p* < 0.05) (Figure 4B). The neuroprotective effects by overexpressed HIF-1α/miR-128-3p were reversed by overexpressed *Axin1*. Thus, upregulated HIF-1α/miR-128-3p or silenced *Axin1* exerted inhibitory effects on apoptosis in hippocampal neurons from MPTP-lesioned mice.

### Overexpression of HIF-1α ameliorates behavioral disorders and pathological changes in MPTP-lesioned mouse model of PD

**Figure 5:**
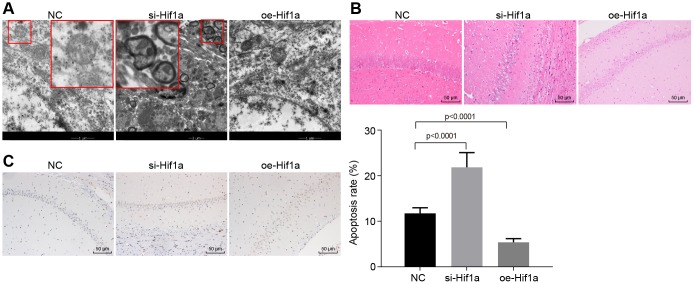
**The pathological changes of hippocampal neurons in the MPTP-lesioned mouse model of PD.** (**A**) The ultrastructure of hippocampal neurons treated with *si-Hif1a* or *oe-Hif1a* to electron microscopy. (**B**) The pathological changes of hippocampal neurons treated with *si-Hif1a* or *oe-Hif1a* revealed by HE staining (scale bar = 50 μm). (**C**) The apoptosis of hippocampal neurons treated with *si-Hif1a* or *oe-Hif1a* detected by TUNEL staining (scale bar = 50 μm). * *p* < 0.05 *vs.* the NC group (MPTP-lesioned mice treated with NC plasmids). N = 10.

The effect of HIF-1α on the progression of mince with PD was evaluated *in vivo*. As shown in Table 1, the MPTP-lesioned mice treated with *oe-Hif1a* showed increased locomotion, standing, and scores for the swimming test and decreased time in the pole climbing test compared to untreated MPTP-lesioned mice (*p* < 0.05). Moreover, electron microscopy showed intact ultrastructural morphology of hippocampal neurons, which were clear structure, with normal nuclear morphology, and uniformly distributed chromatin in mice treated with *oe-Hif1a*, while varying degrees of degeneration of hippocampal neurons, severe lesions, irregular nuclear morphology, lobulated hollow on nuclear membrane, shrink of chromatin within nuclear, and early apoptotic changes were detected in mice treated with *si-Hif1a* (Figure 5A). Additionally, HE stained sections showed uniformly distribution neurons with clear nuclear structure in mice treated with *oe-Hif1a*, while neurons were swollen and had interstitial edema, nuclear pyknosis, eosinophilic degeneration of cytoplasm in brain sections from mice treated with *si-Hif1a* (Figure 5B). Finally, TUNEL staining showed relatively fewer brown-colored positive cells in MPTP-lesioned mice treated with *oe-Hif1a* and more positive cells in those treated with *si-Hif1a* (Figure 5C). Furthermore, apoptosis of hippocampal neurons was increased in mice treated with *si-Hif1a*, but decreased in those treated with *oe-Hif1a* (*p* < 0.05), thus confirming that restored HIF-1α could ameliorate behavioral disorders and neuropathological changes in MPTP-lesioned mice, while silenced HIF-1α exerted the reverse effects.

**Table 1:** Restoration of HIF-1α ameliorates behavioral disorders in MPTP-lesioned mouse model of PD

**Groups**	**Counting of autonomic activities**		**Pole climbing test**	**Swimming test**
	**Distance of movement (lattice)**	**Times of standing**	**Total time**	**Score**
NC	75.40 ± 7.79	18.40 ± 1.96	20.27 ± 1.45	1.30 ± 0.26
si-*Hif1a*	42.00 ± 5.46*	10.50 ± 1.18*	28.64 ± 3.32*	0.40 ± 0.52*
oe-*Hif1a*	127.50 ± 13.95*	32.70 ± 4.55*	10.35 ± 0.92*	2.30 ± 0.42*

Note: * *p* < 0.05 *vs.* the NC group (PD mouse model treated with NC plasmids). N = 10. The measurement data were expressed as mean ± standard deviation. The experiment was repeated 3 times independently.

### Overexpression of HIF-1α upregulates miR-128-3p and downregulates *Axin1* to activate the Wnt/β-catenin signaling pathway in MPTP-lesioned mouse model of PD

**Figure 6:**
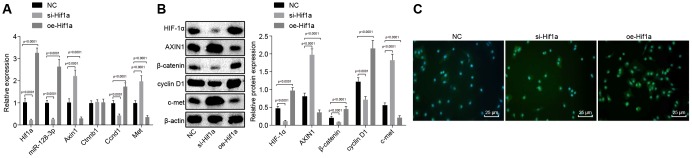
**The expression pattern of HIF-1α/miR-128-3p/Axin1 and the Wnt/β-catenin signaling pathway-related proteins (β-catenin, cyclin D1 and c-met) in hippocampal neurons of the MPTP-lesioned mouse model of PD after transfection with *si-Hif1a* or *oe-Hif1a*.** (**A**) The miR-128-3p expression and mRNA levels of *Hif1a, Axin1, Ctnnb1, Ccnd1,* and *Met* in hippocampal neurons of the MPTP-lesioned mouse model of PD determined by RT-qPCR. (**B**) The protein band patterns and the protein levels of HIF-1α, AXIN1, β-catenin, cyclin D1, and c-met normalized to β-actin in hippocampal neurons of the MPTP-lesioned mouse model of PD determined by western blot analysis. (**C**) The localization of β-catenin protein in hippocampal tissues of the MPTP-lesioned mouse model of PD detected by the immunofluorescence assay (scale bar = 25 μm). * *p* < 0.05 *vs.* the NC group (hippocampal neurons of the MPTP-lesioned mouse model of PD treated with NC plasmids). N = 10.

With the findings that HIF-1α affects behavioral disorders and pathological changes in MPTP-lesioned mice, we next sought to examine how HIF-1α regulates Wnt/β-catenin signaling pathway by modulating miR-128-3p and *Axin1*. RT-qPCR (Figure 6A) revealed elevated mRNA levels of *Axin1* and *Met* and reduced miR-128-3p expression and mRNA levels of *Hif1a*, and *Ccnd1* in hippocampal neurons of MPTP-lesioned mice treated with *si-Hif1a* (*p* < 0.05), while the results were opposite for mice treated with *oe-Hif1a* (*p* < 0.05), where *Ctnnb1* mRNA level was unaffected (*p* > 0.05). Next, western blot analysis (Figure 6B) of hippocampal neurons of MPTP-lesioned mice treated with *si-Hif1a* showed elevated protein levels of AXIN1 and c-met and reduced protein levels of β-catenin, HIF-1α, and cyclin D1, whereas HIF-1α overexpression resulted in the opposite effects (*p* < 0.05). Immunofluorescence assays revealed decreased nuclear translocation in hippocampal neurons of MPTP-lesioned mice treated with *si-Hif1a*, but increased nuclear translocation in mice treated with *oe-Hif1a* (Figure 6C), thus confirming that HIF-1α restoration could promote activation of the Wnt/β-catenin signaling pathway by upregulating miR-128-3p and downregulating *Axin1* in MPTP-treated mice.

## DISCUSSION

Recent years have seen the advent of effective technologies such as molecular imaging for the early diagnosis of PD, but available treatments are only symptomatic. Obtaining a deeper understanding of the complexity of the disease is still required to provide the basis for fundamentally better diagnostic and therapeutic methods for PD [26]. miRNAs can affect PD progression by regulating dopaminergic neurons in the substantia nigra in a PD mouse model [27]. Moreover, the critical impacts of HIF-1α on PD has attracted considerable attention [28]. In this study, we aimed to explore the effect of HIF-1α/miR-128-3p on hippocampal neurodegeneration in PD.

Our initial results demonstrated that HIF-1α and miR-128-3p were downregulated in hippocampus of the PD model mice, while *Axin1* was highly expressed. HIF-1α is a major transcription factor, which is known to be involved in the pathogenesis of PD, and an elevated HIF-1α level can play a neuroprotective roles in a PD model [11]. Notably, our *in vitro* data showed a general correspondence of HIF-1α gene and protein expression levels. HIF-1α has been documented to mediate various genes at the transcriptional level by diverse cofactors [29]. Besides, baseline expression of HIF-1α is epigenetically mediated epigenetically according to t cell type and stage of differentiation [30], providing background to validate our present results. A recent study has proved that HIF-1α can upregulate miR-128 [10] and expression of miR-128 occurs in models of neurodegenerative disorder [31]. Moreover, loss of miR-128 in neurons is found in mice with a PD-like syndrome [9]. Additionally, present findings confirmed that miR-128-3p could target *Axin1* to negatively regulate its expression. AXIN1 is regarded as a multi-domain scaffold protein that combines with various protein complexes, which modulate the Wnt signaling pathway [32]. An existing literature has confirmed that *Axin1*, which is upregulated in PD, is negatively regulated by miR-128, and that miR-128 thus functions as a novel biomarker for PD as well as a potential avenue for treatment by inhibiting *Axin1* [21]. Besides, the vitamin D receptor has been reported to be a transcription regulator for AXIN1, suggesting that AXIN1 can indeed be mediated by transcription [33]. In the current investigation, AXIN1 was highly expressed while HIF-1α, a transcription factor, was poorly expressed in the mouse model of PD, indicating that AXIN1 may be mediated by transcription factors in a direct manner, which is a topic for future research.

Further exploration revealed that overexpressed HIF-1α/miR-128-3p in the MPTP-lesioned mouse model of PD exerted inhibitory effects on hippocampal neuron apoptosis through down-regulation of *Axin1*, by which the suppressive action on the activation of the Wnt/β-catenin signaling pathway was weakened. miRNAs are now understood to participate in multiple physiological processes, including cell proliferation, differentiation, and apoptosis [34]. miR-128 overexpression in neurons rescues mice from motor deficits in a PD model [9]. Moreover, HIF-1α upregulates a great number of target genes that modulate cell cycle distribution [11], and increased expression of HIF-1α protects neurons against degeneration in a PD model [35]. It is well known that HIF-1α regulates the Wnt/β-catenin signaling pathway and that β-catenin protects neurons against misfolded protein-mediated disorders (including PD) by preventing mitochondrial malfunction [12]. Abnormal expression of miRNAs can activate several signaling pathways, including the Wnt/β-catenin signaling pathway [36], which can inhibit neurodegeneration by regulating cell death [37]. The activation of the Wnt signaling pathway can exert protective effects on hippocampal neurons in a PD model [14]. Furthermore, AXIN1 inhibits activation of the Wnt/β-catenin signaling pathway, which leads to degeneration of hippocampal neurons [22], suggesting that silenced *Axin1* could alleviate hippocampal neurodegeneration by activating the Wnt/β-catenin signaling pathway. Additionally, miR-128-3p can promote activation of the Wnt/β-catenin signaling pathway [38], whereas Baldeschi et al. have revealed that dysregulation of the Wnt/β-catenin signaling pathway is implicated in PD [39]. These findings support our contention that up-regulation of HIF-1α/miR-128-3p axis could suppress apoptosis of hippocampal neurons in MPTP-lesioned mice via inhibition of *Axin1* and activation of the Wnt/β-catenin signaling pathway. Meanwhile, the *in vivo* experiments in our study also demonstrated that overexpression of HIF-1α could ameliorate motor symptoms and pathological changes in MPTP-lesioned mice. Li et al. have proved that restored HIF-1α exerts neuroprotective effects with potential for treatment of PD [11]. What’s more, in hippocampal neurons of MPTP-lesioned mice *in vivo*, elevated HIF-1α led to up-regulation of miR-128-3p, and down-regulation of *Axin1*, thus reducing the inhibition of Wnt/β-catenin signaling pathway. Notably, the potential value of activating HIF-1 has been highlighted in consideration of its positive and protective effects in neurodegenerative models, predicting that it might likewise interfere with pathogenesis of idiopathic PD and ameliorate the clinical outcomes of PD patients [40].

In conclusion, we have proved that activated HIF-1α/miR-128-3p axis inhibits hippocampal neurodegeneration in a MPTP-lesioned mouse model of PD via activation of the Wnt/β-catenin signaling pathway through targeting *Axin1*. Thus, elevated HIF-1α/miR-128-3p may present promising new target for developing therapeutic treatments for patients with PD. Ongoing preclinical research should eventually establish prospects for clinical translation of this approach to disease modifying treatment of PD.

## MATERIALS AND METHODS

### Microarray analysis

The PD-related microarray dataset (GSE7621) and its annotation probe file were obtained from the Gene Expression Omnibus (GEO) database (https://www.ncbi.nlm.nih.gov/geo/), and packages in the R software were used to conduct the differential analysis. The differential expressed genes (DEGs) were screened with the threshold *p* value < 0.05, and the heatmaps of DEGs were drawn.

### Experimental animals

A total of 40 healthy C57BL/6J male mice (weighing 18–22 g and aged eight weeks) were purchased from the Shanghai Institute of Pharmaceutical Industry (Shanghai, China). All mice were reared in a laminar flow rack without special pathogen control at a constant temperature of 24–26ºC and relative humidity of 45%–55%. Food and drinking water were available ad libitum after high-temperature sterilization.

### Establishment of PD mouse model

After dissolving in aseptic saline, the 1-methyl-4-phenyl-1,2,3,6-tetrahydropyridin (MPTP) (25 mg/Kg, Sigma-Aldrich Chemical Company, St Louis, MO, USA) was administered to the mice by intraperitoneal injection once a day for seven days to establish the the PD mouse model. Control mice were injected with an equivalent volume of saline [41].

### Animal grouping and behavioral experiments

The mice were anaesthetized with intraperitoneal injection of 2% sodium pentobarbital (40 mg/kg). The skin of the head was disinfected using alcohol, and the scalp was cut in a longitudinal direction to expose the skull. A small diameter hole was drilled 1 mm behind of Bregma and 2 mm later to the midline using a #5 needle. The medium containing lentivirus (10 μL, titer 2 × 10^8^ U/mL) was injected into the mice using a microsyringe, which was inserted vertically to a depth of about 2 mm. After completing the injection, the needle remained in place for 5 min to allow diffusion of the lentivirus prior to withdrawal.

On the 25^th^ day after the model establishment, the *in vivo* transfection was conducted as described above. A total of 30 model mice were transduced with blank control lentivirus, *Hif1a* silencing lentivirus, or *Hif1a* overexpression lentivirus. Two days later, we measured autonomic activity [42], and performed the pole climbing test [43] and swimming test [44] as described previously. Finally, the mice were euthanized, and the brain tissues were collected for the subsequent analyses.

### Isolation of primary hippocampal neurons from mice

After brain removal, the hippocampus and cerebral cortex were separated on ice, cut into blocks of 0.5–1 mm^3^, and suspended into single cell suspension by mechanical homogenization in buffer. The cells were cultured in the Dulbecco's Modified Eagle's Medium (DMEM, Gibco, Carlsbad, CA, USA) supplemented with 10% horse serum (Gibco, Carlsbad, CA, USA), 5% fetal bovine serum (FBS, Gibco, Carlsbad, CA, USA), and 0.1% glutamine (Sigma-Aldrich Chemical Company, St Louis, MO, USA) and DMEM culture medium containing 10% horse serum, 0.1% glutamine (Sigma-Aldrich Chemical Company, St Louis, MO, USA), 1% ITS (Sigma-Aldrich Chemical Company, St Louis, MO, USA), 0.01% E_2_ (Sigma-Aldrich Chemical Company, St. Louis, MO, USA), and NaHCO_3_ (0.12 g/100 mL). The tissues were rinsed twice with Hank buffer (Thermo Fisher Scientific, Waltham, MA, USA), cut into blocks (0.5–1 mm^3^) and detached by 0.125% trypsin-ethylenediaminetetraacetic acid at 37°C for 5 min. The detachment was terminated by adding DMEM feeding solution. The cells were then plated into polylysine-coated culture dishes (Sino-American Biotechnology Co., Ltd., Beijing, China) at a density of 1 – 5 × 10^5^ cells/mL and incubated with 5% CO_2_ at 37°C. After three days, cytarabine (Sino-American Biotechnology Co., Ltd., Beijing, China) was added to the medium to inhibit the growth of glial cells. DMEM culture medium was added 24 h later. The medium was renewed after 3 – 5 days and mouse primary hippocampal neurons were obtained.

### Cell grouping and transfection

The amplified sequences of *Hif1a*, *Axin1* and miR-128-3p were separately incorporated into the GV287 or GV248 carriers (Shanghai Genechem Co., Ltd., Shanghai, China). GV287-based lentiviral vectors (Shanghai Genechem Co., Ltd., Shanghai, China) were transduced to construct *Hif1a*-, *Axin1*- and miR-128-3p-overexpressed the normal hippocampal neurons or hippocampal neurons in the context of PD. In addition, the GV248-based lentiviral vectors (Shanghai Genechem Co., Ltd., Shanghai, China) incorporated with siRNAs against *Hif1a*, *Axin1* and miR-128-3p were constructed and transduced into the normal and PD model hippocampal neurons. Lentiviral transduction was performed with fivefold multiplicity of infection, according to the manufacturer’s instructions. The seeded 6-well plates were placed in an incubator at 37 °C with 5% CO_2_ for 24 h for subsequent experiments.

Cells were divided into 2 parts and the detailed treatment modalities were as follows: (1) The primary hippocampal neurons from normal mice were transduced with blank lentivirus vector, lentivirus vector harboring siRNA against *Hif1a* (*si-Hif1a*), miR-128-3p inhibitor, lentivirus vector harboring overexpression plasmid of *Axin1* (oe-*Axin1*), lentivirus vector harboring *si-Hif1a* + siRNA against *AXIN1* (*si-Axin1*), miR-128-3p inhibitor + lentivirus vector harboring *si-Axin1* or lentivirus vector harboring *si-Hif1a* + miR-128-3p inhibitor + lentivirus vector harboring *si-Axin1*. (2) The primary hippocampal neurons from MPTP-lesioned mouse model of PD were transduced with blank lentivirus vector, lentivirus vector harboring *oe-Hif1a*, miR-128-3p mimic, lentivirus vector harboring *si-Axin1*, lentivirus vector harboring *oe-Hif1a* + oe-*Axin1*, miR-128-3p mimic + lentivirus vector harboring oe-*Axin1* or lentivirus vector harboring *oe-Hif1a* + miR-128-3p mimic + lentivirus vector harboring oe-*Axin1*.

### RNA isolation and quantitation

The total RNA was extracted from the hippocampal tissues of mouse brains and hippocampal neurons using Trizol (Invitrogen, Carlsbad, CA, USA). After determination of RNA concentration using Nanodrop 2000 (Thermo Fisher Scientific Inc., Waltham, MA, USA), cDNA synthesis and reverse transcription (RT) of antisense miRNA were conducted using a polymerase chain reaction (PCR) amplification instrument (Thermo Fisher Scientific Inc., Waltham, MA, USA) The RT quantitative PCR (RT-qPCR) was performed on the ABI7500 qPCR instrument (Thermo Fisher Scientific Inc., Waltham, MA, USA) with β-actin and U6 as the internal references. The 2^-ΔΔCt^ method was adopted to analyze the relative expression of target genes [45]. The primer sequences are shown in Table 2. The primers were designed and synthesized by the Shanghai GenePharma Co., Ltd. (Shanghai, China).

**Table 2:** Primer sequences for RT-qPCR.

**Gene**	**Primer sequence**
*Hif1a*	Forward 5’-TCGAAGTAGTGCTGATCCTGC-3’
	Reverse 5’-GGCTGGGAAAAGTTAGGAGTGT-3’
*Axin1*	Forward 5’-GGACCTCGGAGCAAGTTTCA-3’
	Reverse 5’-GGTTGACAGGCCTCGAATCA-3’
*Ctnnb1*	Forward 5’-CCATGATCCGGGCCTTCTC-3’
	Reverse 5’-GTACTCCTGCCTCTCTCGGA-3’
*Ccnd1*	Forward 5’-GCCTGCCAGGAACAGATTGA-3’
	Reverse 5’-TTTGCGGGTGCCACTACTT-3’
*Met*	Forward 5’-GGAACTATGACCTCGACTACGAC-3’
	Reverse 5’-ACCATGTCTCCTACAGTACCTC-3’
*Actb*	Forward 5’-GAACCCTAAGGCCAACCGTGAA-3’
	Reverse 5’-CTCAGTAACAGTCCGCCTAGAA-3’
*miR-128-3p*	Forward 5’-GGTCACAGTGAACCGGTC-3’
	Reverse 5’-GTGCAGGGTCCGAGGT-3’
*U6*	Forward 5’-CTCGCTTCGGCAGCACA-3’
	Reverse 5’-AACGCTTCACGAATTTGCGT-3’

Note: RT-qPCR, reverse transcription quantitative polymerase chain reaction; HIF-1α, hypoxia inducible factor-1α, miR-128-3p, microRNA-128-3p.

### Western blot analysis

The total protein was extracted from the hippocampal tissues of mouse brains and hippocampal neurons by the protein lysis buffer (Beyotime Institute of Biotechnology, Shanghai, China). Portions of the extracted proteins (30 μg) were separated by sodium dodecyl sulfate-polyacrylamide gel electrophoresis, and transferred onto a polyvinylidene fluoride membrane (Amersham, NJ, USA). The membrane was incubated at 4ºC overnight with mouse anti-HIF-1α monoclonal antibody (1:500, ab1), rabbit anti-AXIN1 polyclonal antibody (1:1000, ab55906), rabbit anti-β-catenin monoclonal antibody (1:5000, ab32572), rabbit anti-cyclin D1 monoclonal antibody (1:200, ab16663), rabbit anti-c-met monoclonal antibody (1:1000, ab51067), and mouse anti-β-actin monoclonal antibody (1:5000, ab8225). The abovementioned antibodies were purchased from Abcam Inc. (Cambridge, MA, USA). After 3 rinses with tris-buffered saline containing 0.1% Tween-20 three times (10 min per rinse), the membrane was incubated with the horseradish peroxidase-labeled goat anti-mouse or goat anti-rabbit secondary antibody (1:10000, Jackson Immunoresearch Laboratories, West Grove, PA, USA). The Image Pro Plus 6.0 (Media Cybernetics, Silver Springs, MD, USA) software was utilized to analyze the protein levels through grayscale scanning of protein bands.

### Immunofluorescence assay

The mouse hippocampal tissue sections were incubated with the rabbit anti-β-catenin antibody (1:250, ab32572) at 4°C overnight, and washed three times with phosphate buffer saline (PBS; 5 min per rinse). Next, the sections were incubated with fluorescein isothiocyanate (FITC)-labelled goat anti-rabbit secondary antibody (1:1000, ab6717) at 37°C for 1 h in subdued light, followed by washing with PBS and incubation with 4', 6-diamidino-2-phenylindole at 37°C for 1 h in the dark. Five randomly selected fields were observed and photographed under the laser scanning confocal microscope (Olympus, Tokyo, Japan). The antibodies above were purchased from Abcam Inc. (Cambridge, MA, USA).

### Dual-luciferase reporter gene assay

The target gene of miR-128-3p was predicted by Targetscan at the biological prediction website (http://www.targetscan.org/vert_71/), and the putative binding sites between miR-128-3p and 3'untranslated region (3’UTR) of *Axin1* were verified by dual-luciferase reporter gene essay. *Axin1* 3'UTR gene fragments were synthesized *in vitro* and introduced into the pMIR-reporter (Promega, Madison, WI, USA). The complementary sequence mutation sites of seed sequences were designed based on the wild type (WT) of *Axin1*. The WT or mutant (MUT) target fragments were inserted into the pMIR-reporter plasmid. These two reporter vectors were respectively co-transfected with miR-128-3p mimic and pRL-TK into HEK-293T cells (Shanghai Beinuo Biotechnology Co., Ltd., Shanghai, China). The luciferase activity was measured using the Dual-Luciferase Reporter Assay System kit (Promega Corporation, Madison, WI, USA) on the fluorescence detector (Promega Corporation, Madison, WI, USA). The ratio of the activity of Firefly luciferase to that of Renilla luciferase was regarded as the relative luciferase activity.

### Annexin V/propidium iodide (PI) staining

The hippocampal neurons were washed with PBS and detached with 0*.*25% trypsin. The suspended cells were separated by centrifugation at 1000 r/min for 5 min. Subsequently, the cells were washed with cold PBS three times, and the cell concentration was adjusted to 10^6^ cells/mL. The cells were resuspended with 500 μL binding buffer, followed by incubation with 5 μL Annexin-V-FITC and 5 μL PI (Sigma-Aldrich Chemical Company, St Louis, MO, USA) at room temperature in subdued light for 15 min. Apoptosis of hippocampal neurons was detected by the flow cytometer within 1 h of treatment.

### Ultrastructural observation

The hippocampal tissue sections were immersed in the solution prepared with Epon8l2 embedding resin mixed with acetone (3:1) at 35°C for 45 min, and then immersed in Epon8l2 embedding resin at 45°C for 2 h. After polymerization, the Epon8l2-embedded sections were sliced using a microtome (Leica, Wetzlar, Germany) and stained with uranium acetate and lead citrate. The ultrastructural changes were observed and photographed under the H-600IV transmission electron microscope (Hitachi, Tokyo, Japan).

### Hematoxylin and Eosin (HE) staining

The hippocampal tissue sections were paraffin-embedded before staining with hematoxylin (Beyotime Institute of Biotechnology, Shanghai, China) for 5 min, and then with eosin (Beyotime Institute of Biotechnology, Shanghai, China) for 2 min. The stained sections were observed and photographed under an optical microscope (DMI3000, Leica, Wetzlar, Germany).

### TUNEL staining

Apoptosis of hippocampal tissues was observed using the TUNEL kit (Beyotime Institute of Biotechnology, Shanghai, China) according to the manufacturer´s instructions. The paraffin-embedded tissue slices (5 μm) were exposed with 50 μL of TUNEL solution (Thermo Fisher Scientific, Waltham, MA, USA) for 1 h at 37°C and incubated with diaminobenzidine (ZSGB-BIO, China) for 10 min. After counter-staining with hematoxylin, the sections were observed and photographed under the microscope (Nikon, Japan). Normal cells and TUNEL positive cells were counted in five randomly selected fields. The cell apoptotic rate was calculated as the average number of positive stained nuclei/total number of nuclei × 100%.

### Statistical analysis

Statistical analysis was performed using the SPSS 21.0 software (IBM Corp. Armonk, NY, USA), and the measurement data were expressed as mean ± standard deviation. Comparison of data with normal distribution and homogeneity of variance between two groups was analyzed using unpaired *t*-test. Comparison among multiple groups was tested by one-way analysis of variance (ANOVA), followed by Tukey’s *post hoc* test. *p* < 0.05 was considered to be statistically significant.

### Ethics statement

All animal experiments in this study were approved by the Ethics Committee of The Third Affiliated Hospital of Qiqihar Medical University.

## Supplementary Material

Supplementary Figure 1
