# Supplementary material for: HIF-1α/microRNA-128-3p axis protects hippocampal neurons from apoptosis via the Axin1-mediated Wnt/β-catenin signaling pathway in Parkinson’s disease models
Source: Aging (Albany NY). 2020 Mar 12;12(5):4067–81. doi: 10.18632/aging.102636 (PMC7093183; doi:10.18632/aging.102636)
Supplement: Supplementary Figure 1 [file aging-12-102636-s001..pdf]

SUPPLEMENTARY FIGURE

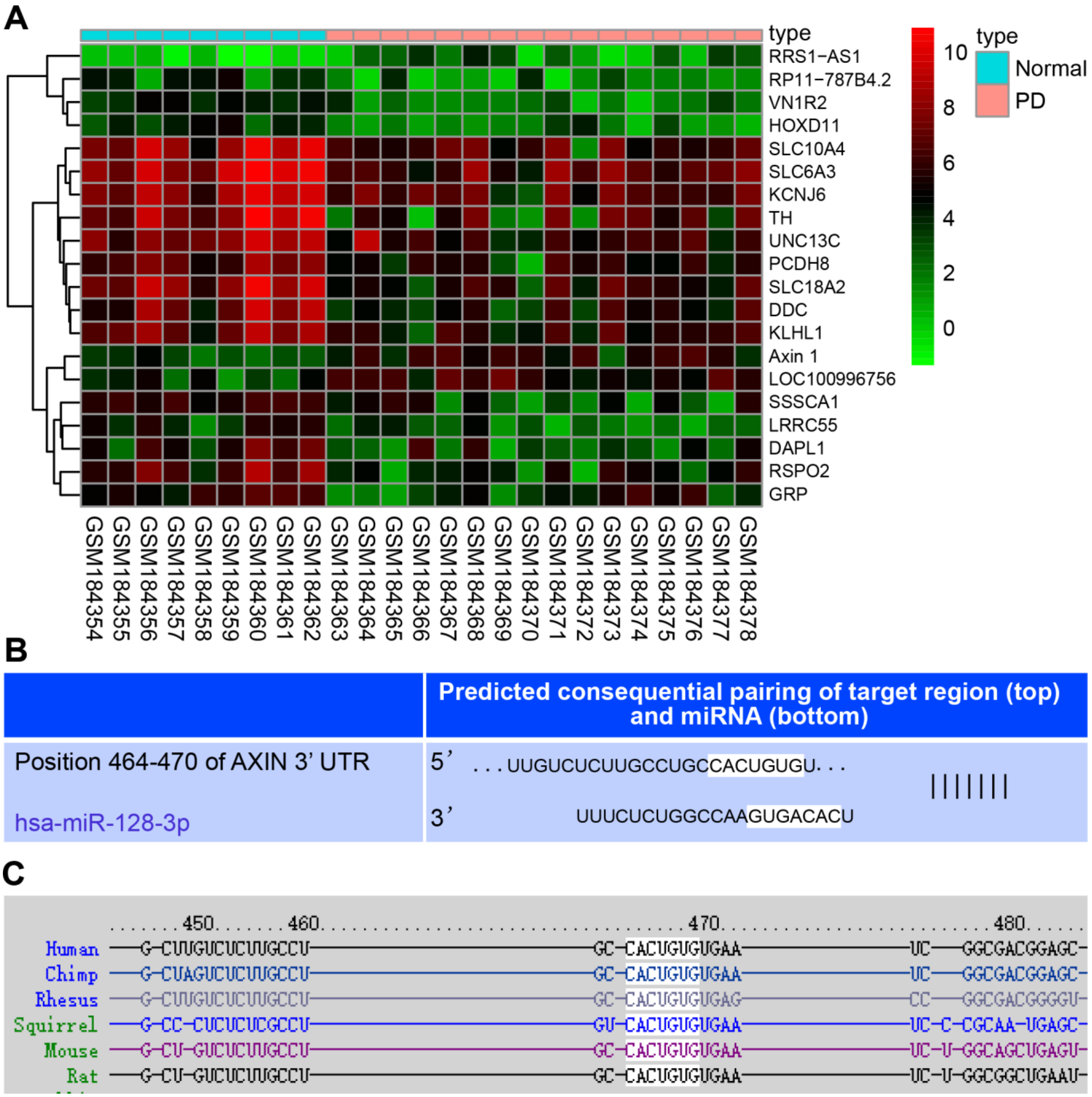

Supplementary Figure 1. The therapeutic significance of the HIF-1α/miR-128-3p/AXIN1 axis in hippocampal neurons via the Wnt/β-catenin signaling pathway. (**A**) The heatmap of DEGs that were screened from the PD-related microarray dataset GSE7621; the abscissa represents the sample number, and the ordinate represents the DEGs; the clustering of gene expression level is presented using a dendrogram in the upper left region, each rectangle indicates the expression of one gene in one sample, and the color gradation is presented using a histogram in the upper right region. (**B**) The predicted binding sites between miR-128-3p and *Axin1* 3'UTR using Targetscan. (**C**) The sequence homology between human and mouse miR-128-3p and *Axin1* 3'UTR.
